# Prenatal dexamethasone and postnatal high-fat diet have a synergistic effect of elevating blood pressure through a distinct programming mechanism of systemic and adipose renin–angiotensin systems

**DOI:** 10.1186/s12944-018-0701-0

**Published:** 2018-03-14

**Authors:** Hong-Ren Yu, You-Lin Tain, Mao-Meng Tiao, Chih-Cheng Chen, Jiunn-Ming Sheen, I-Chun Lin, Shih-Wen Li, Ching-Chou Tsai, Yu-Ju Lin, Kai-Sheng Hsieh, Li-Tung Huang

**Affiliations:** 1 Department of Pediatrics, Chang Gung Memorial Hospital-Kaohsiung Medical Center, #123, Ta-Pei Road, Niao-Sung District, Kaohsiung, Taiwan; 2 Graduate Institute of Clinical Medical Science, Chang Gung University College of Medicine, Kaohsiung, Taiwan; 3 Department of Obstetrics and Gynecology, Chang Gung Memorial Hospital-Kaohsiung Medical Center, #123, Ta-Pei Road, Niao-Sung District, Kaohsiung, Taiwan

**Keywords:** Prenatal glucocorticoid, High-fat diet, Adipose tissue, Renin–angiotensin system

## Abstract

**Background:**

Hypertension may result from high-fat (HF) diet induced-obesity and overexposure to glucocorticoids in utero. Recent studies demonstrated the potent contribution of adipose tissue’s renin-angiotensin system (RAS) to systemic RAS, which plays a key role in regulating blood pressure (BP). In this study, we investigated the effects of prenatal dexamethasone (DEX) exposure and postnatal HF diet on RAS of adipose tissue.

**Methods:**

RAS and BP of 6-month old rats exposed to prenatal DEX and/or postnatal HF diet were examined.

**Results:**

Prenatal DEX plus postnatal HF exerted a synergistic effect on systolic BP. Prenatal DEX exposure suppressed plasma angiotensin (ANG) I and ANG II, whereas postnatal HF suppressed plasma ANG-(1–7) level. Prenatal DEX increased prorenin receptor and renin levels, but suppressed angiotensinogen (AGT) and angiotensin-converting-enzyme 1 (ACE1) mRNA expressions in adipose tissue. Postnatal HF increased AGT mRNA expression, but suppressed prorenin receptor, renin, ACE2, ANG II type 2 receptor (AT2R), and Mas receptor (MasR) mRNA expression levels.

**Conclusions:**

Prenatal GC exposure altered the ACE1/ANG II/ANG II type 1 receptor (AT1R) axis, whereas postnatal HF negatively impacted the ACE2/ANG-(1–7)/MasR axis. Prenatal DEX exposure and postnatal HF synergistically elevated BP through a distinct programming mechanism of systemic and adipose RAS. Adipose RAS might be a target for precise hypertension treatment.

**Electronic supplementary material:**

The online version of this article (10.1186/s12944-018-0701-0) contains supplementary material, which is available to authorized users.

## Background

There is a strong association between obesity and hypertension. It is estimated that 65 to 75% of primary hypertension is related to obesity [1]. High-fat/high-sugar western diet and less physical activity of the modern life style result in higher energy intake and lower energy expenditure. Obesity can lead to kidney injury in addition to hypertension. The mechanism underlying the development of obesity associated hypertension is as follows: (1) physical compression of the kidneys by intrarenal fat and extracellular matrix; (2) impairment of pressure natriuresis; (3) renin–angiotensin system (RAS) activation; (4) increase in the activity of sympathetic nervous system; (5) insulin resistance; and (6) leptin, and other neuropeptide dysregulation [2, 3]. Individuals with obesity are often reported to have increased plasma renin and angiotensin converting enzyme (ACE) activities, plasma angiotensinogen (AGT), angiotensin II (ANG II), or aldosterone levels [2, 4, 5].

Hypertension might have its origins in utero [6]. During human pregnancy, if preterm delivery is expected, glucocorticoid (GC) treatment is given to stimulate the development of the fetal lungs and decrease the postnatal mortality and morbidity [7–9]. Overexposure to GC has been observed in conditions of prenatal stress, which can lead to the developmental programming of metabolic syndrome and hypertension in later life [10–13]. Our previous studies support these adverse effects of prenatal GC over-exposure during fetal development [12, 14–17]. We also showed that dexamethasone (DEX) treatment during pregnancy enhances the susceptibility of the offspring to postnatal HF diet-induced programmed hypertension [18]. The causes for the induction of hypertension by prenatal DEX may include a decrease in the number of nephrons and altered levels of luminal ANG II and sodium transporter [13, 19, 20].

The RAS is classically considered to play a key role in regulating blood pressure (BP) as well as maintaining water and sodium balance. The juxtaglomerular apparatus in the kidney converts prorenin into renin, which is then secreted into circulation. Plasma renin converts AGT into angiotensin I (ANG I). ANG I can be further cleaved by angiotensin-converting-enzyme 1 (ACE1) to yield ANG II. ANG II exerts its effects via binding to the ANG II type 1 receptor (AT1R) and ANG II type 2 receptor (AT2R) [21]. Regulation of RAS is also influenced by other RAS members, such as ANG-(1–7) [22]. Angiotensin-converting-enzyme 2 (ACE2), a homolog of classic ACE1 forms ANG-(1–7) directly from ANG II and indirectly from ANG I. Mas receptor (MasR) is the ANG-(1–7)-specific G-protein–coupled receptor [23]. The ACE2/ANG-(1–7)/MasR axis functions against the vasoconstrictor, proliferative, and lipogenesis effects of ACE1/ANG II/AT1R axis [24]. In addition to BP regulation, RAS is involved in other physiologic and pathophysiologic processes such as growth and inflammation, and involved in glucose and lipid metabolism [21].

Adipose tissue has been reported to express all the RAS components, including AGT, ANG, renin, and AT1R and AT2R [25]. In rodent models, up to 30% of the systemic circulating AGT level was contributed by the adipose tissue [26]. An earlier study showed that in subjects with obesity related hypertension, *AGT* gene expression in adipose tissue was increased and plasma AGT levels were elevated [27]. In this study, we investigated the effects of prenatal DEX exposure and post-natal HF diet on RAS signaling in adipose tissue. We found prenatal GC exposure altered the ACE1/ANG II/AT1R axis, whereas postnatal HF diet negatively impacted the ACE2/ANG-(1–7)/MasR axis. Thus, prenatal DEX exposure and postnatal high-fat diet exerted a synergistic effect on blood pressure elevation through a distinct programming mechanism of systemic and adipose RAS.

## Methods

### Animals

This study was performed in accordance with the Guide for the Care and Use of Laboratory Animals of the National Institutes of Health and was approved by the Institutional Animal Care and Use Committee of Chang Gung Memorial Hospital Kaohsiung Medical Center. Virgin Sprague-Dawley (SD) rats (approximately 12–16 weeks old) were obtained from BioLASCO Taiwan Co., Ltd. (Taipei, Taiwan), housed and maintained in a facility certified by the Association for the Assessment and Accreditation of Laboratory Animal Care International. The study protocol was described earlier [28]. In brief, the virgin SD female rats were allowed to mate with male rats for 24 h, and were then separated from the male rats and housed individually in standard plastic home cages. After confirmation of pregnancy, the pregnant female rats were randomly assigned to two groups: the vehicle group and dexamethasone exposure group. The animals in the dexamethasone group were injected intraperitoneally with 0.1 mg/kg/day dexamethasone every day from the gestational age of 14–20 days. The vehicle group received daily intraperitoneal injections of normal saline during the same period. The subjects from litters of eight pups were standardized with respect to the quantity of milk they received and maternal pup care after birth. The offspring were allowed to stay with the mother until weaning. Pups were weaned 21 days after birth and placed in cages in groups of three until testing in adulthood. Since hypertension occur at a higher rate than females, we elected to study only male offspring [29, 30]. The male offspring were then divided into four groups: the VEH group, prenatal DEX exposure group, postnatal high-fat diet group (VHF), and prenatal dexamethasone exposure with postnatal high-fat diet group (DHF) (*n* = 8–10 for each group). VEH and DEX groups received control diet (protein 23.5%, fat 4.5%, crude fiber 5.0%, crude ash 7.0%, and water 13%, Fwusow Taiwan Co., Ltd., Taichung, Taiwan) after weaning. VHF and DHF groups received a HF diet (58% high-fat diet, Research Diet, D12331) from weaning to 6 M of age.

### Body weight and blood pressure measurement

The body weights of the offspring were measured every month until 6 months after birth. BP was measured in conscious rats at the age of 6 months using an indirect tail-cuff method (BP-2000, Visitech Systems, Inc., Apex, NC, USA) with 1 week of adaptation before the experiment [31]. In brief, rats were placed on a platform with their tails passed through tail cuffs and secured in place with tape. The rats were allowed to adjust the inflating cuff with 10 preliminary cycles of tail cuff inflation. A total of 5 cycles were recorded at each time point. The average value was calculated from three stable measurements.

### Experimental procedures and specimen collection

Rats were killed at 6 months of age by using exsanguination under anesthesia. Rats were euthanized using xylazine (50 mg/kg; Bayer, Taipei, Taiwan) and zoletil 50® (25 mg/kg; Virbac Laboratories, Carros, France) at a 1:2 mixture by intramuscular injection, followed by cardiac puncture and saline perfusion [28, 32]. Heparinized blood samples were collected at the time of euthanasia [28]. Four regions of adipose depots were carefully dissected as described earlier [33]. In brief, first, the epididymal depot was separated by a horizontal cut above the epididymis, just distal to the major blood vessel in the base of the pad. Second, the retroperitoneal depot was recovered by first separating the perirenal fat and then dissecting the entire retroperitoneal pad as a triangular section extending from a vertex in the inguinal region up the midline and across at the lower pole of the kidney, extending laterally as far as fat was visible. Third, the mesenteric depot was harvested by cutting the intestine below the duodenal-jejunum junction and stripping the fat by gently pulling the intestinal loops apart. Fourth, the retroperitoneal depot was dissected out, laying the animal first on the left and then on the right side. Lateral cuts were made through the skin from the top of the buttock to the base of the ear to reveal the underlying fatty layer. At approximately halfway point of this cut, lateral cuts were made into the fatty pad, following the line of the previous skin incision. Next, a cut was made through the skin just below the rib cage across the dorsal surface linking the two lateral incisions. The rectangular flap of skin was then carefully separated, leaving the subcutaneous pad intact. The pad was then carefully dissected away from the underlying muscle and fascia. The weights of retroperitoneal, epididymal, mesenteric and subcutaneous fats were recorded. Because visceral or retroperitoneal fat is a better predictor of increased BP than subcutaneous fat [34] and retroperitoneal fat has been reported more correlated with metabolic profiles than other fat depots [35]. Thus, retroperitoneal fat was chosen for further RT-PCR studies.

### Determination of plasma ANG I, ANG II, and ANG-(1–7) levels

Plasma ANG I, ANG II, and ANG-(1–7) levels were determined by ELISA (MyBioSource, San. Diego, USA. IMBS742662, MBS269721, and MBS731540, respectively) with adequate dilution as indicated. The detection limits for ANG I, ANG II, and ANG-(1–7) ELISA are 5 pg/ml, 12 pg/ml, and 0.1 ng/ml, respectively with intra assay precision and inter assay precision ≤ 12 according to the manufacturer’s instructions. There is no cross-reaction with each other.

### Reverse transcription (RT)-polymerase chain reaction (PCR)

RT-PCR was performed as previously described [16]. In brief, total RNA was extracted from adipose tissues with Trizol reagent (#15596-018, Invitrogen, Carlsbad, CA). A 5-μg sample of total RNA was reverse transcribed with 200 U of Moloney murine leukemia virus reverse transcriptase (Invitrogen). PCR was performed in 20 μl total reaction volume containing 2 μl 1:10 diluted cDNA, specific primers, 2.5 mM MgCl_2_, and Maxima SYBR Green/Fluorescein qPCR Master Mix (2X) (#K0242, Thermo Scientific, CA, USA). The cycling protocol comprised initial denaturation step of 10 min at 95 °C followed by 45 cycles of denaturation for 10 s at 95 °C, annealing for 20 s at 55 °C, and extension for 20 s at 72 °C. The primers used are shown in Additional file 1: Table S1. The threshold cycles (Ct) were detected with LightCycler software (ver. 1.5.0). Standard curves were then plotted with Ct versus log cDNA quantity, and the sample quantities were estimated from the standard curves. Comparative Ct method was used for relative quantification of gene expression. The averaged Ct was subtracted from the corresponding averaged β-actin value for each sample, resulting in △Ct. △△Ct was calculated by subtracting the average control △Ct value from the average experimental △Ct value. The fold increase was calculated as 2-△△Ct for the experimental vs. control samples.

### Statistics

Differences between two groups were analyzed via Mann-Whitney U test. Two-way ANOVA was used to evaluate the effect of the interaction of prenatal DEX exposure and postnatal HF diet as dependent variables; significant interaction was determined using a subsequent simple-effects analysis with Bonferroni correction. Data were expressed as mean ± standard error of the mean. *p*-value less than 0.05 was considered statistically significant for all tests. All statistical analyses were performed using SPSS 19.0 for Windows XP (SPSS, Inc., Chicago, IL, USA).

## Results

### Both prenatal DEX exposure and postnatal HF diet increased body weight and BP in offspring at 6 M

**Fig. 1:**
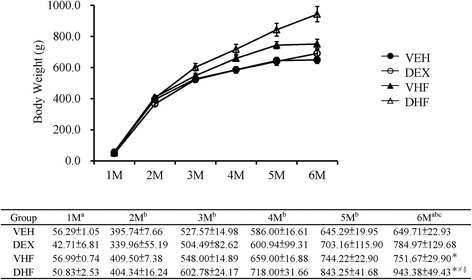
Effects of prenatal dexamethasone (DEX) exposure (DEX), postnatal high-fat (HF) diet (VHF), and prenatal DEX plus postnatal HF diet exposure (DHF) on body weight. Body weight was measured in conscious rats between 1 and 6 months of age. *N* = 8–10/group. Two-way ANOVA was used to evaluate the individual effects of prenatal DEX (Hit 1) and postnatal HF diet (Hit 2) and their interaction effect (Hit 1 x Hit 2). Abbreviations: VEH; vehicle. ^a^Hit 1, *p* < 0.05; ^b^Hit 2, *p* < 0.05; ^c^Hit 1 × Hit 2, *p* < 0.05. *as compared with VEH, *p* < 0.05; ^#^as compared with DEX, *p* < 0.05; ^§^as compared with VHF, *p* < 0.05

In our study, we found there is no difference in the litter size and ratio of male-to-female pups as reported earlier [16]. The DEX group exhibited the lowest body weight (42.71±6.81 g) compared to that of the other groups at 1 M (Fig. 1). Two-way ANOVA between groups showed a significant main effect for prenatal DEX exposure (Hit 1) [F (1, 30)  =  17.18, *p* < 0.001] without prenatal DEX/postnatal HF diet (Hit 2) interaction. Thus, prenatal DEX over-exposure resulted in failure to thrive of offspring. As the offspring grew, two-way ANOVA analysis revealed a significant effect of Hit 2 on offspring body weight with [2 M, F (1, 30)  =  4.54, *p* = 0.041; 3 M, F (1, 30)  =  6.92, *p* = 0.013); 4 M, F (1, 30)  =  19.52, *p* < 0.001; and 5 M, F (1, 30)  =  26.97, *p* < 0.001]. It suggested postnatal HF exposure cause body over-weight since early life. However, at 6 M, main effects were confirmed for Hit 1 [F (1, 29)  =  11.82, *p* =  0.002] and Hit 2 [F (1, 29)  =  27.29, *p* < 0.001]. A positive interactive effect of prenatal DEX exposure and postnatal HF diet on BW [F (1, 29) =  4.91, *p* = 0.035] was confirmed by two-way ANOVA. Thus, postnatal DEX group presented heavier body weight than control group as adult stage, although the BW of the postnatal DEX group was less than control at young infant stage. The DHF group had the highest BW compared to that of other groups. Prenatal DEX plus postnatal HF exerted a synergistic effect on the increase of BW.

**Fig. 2:**
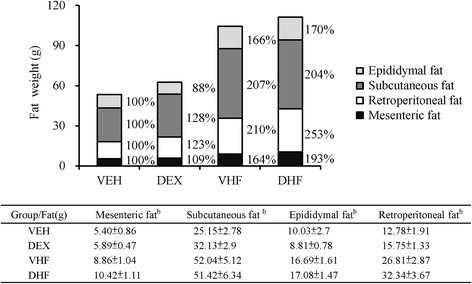
Effects of prenatal DEX exposure (DEX), postnatal HF diet (VHF), and prenatal DEX exposure plus postnatal HF diet (DHF) on the weight of different adipose tissue depots. Weights of different adipose depots were measured at 6 months of age. *N* = 8–10/group. Two-way ANOVA was used to evaluate the individual effects of prenatal DEX exposure (Hit 1) and postnatal HF diet (Hit 2) and their interaction effect (Hit 1 x Hit 2). The percentage shown in the figure was the adipose depot weight relative to that of the vehicle group (VEH). ^a^Hit 1, *p* < 0.05; ^b^Hit 2, *p* < 0.05; ^c^Hit 1 × Hit 2, *p* < 0.05. *as compared with VEH, *p* < 0.05; ^#^as compared with DEX, *p* < 0.05; ^§^as compared with VHF, *p* < 0.05

Two-way ANOVA analysis of adipose tissue weight in different groups at 6 M, demonstrated a significant main effect for postnatal HF diet [mesenteric, F (1, 48)  =  12.32, *p* = 0.001; retroperitoneal, F (1, 48)  =  18.07, *p* < 0.001; subcutaneous, F (1, 48)  =  13.41, *p* = 0.001; and epididymal, F (1, 48)  =  15.23, *p* = 0.001] without Hit 1/Hit 2 interactions (Fig. 2). Postnatal HF showed a statistic significantly effect on the increase of adipose tissue weight.

**Fig. 3:**
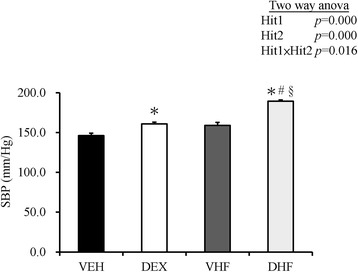
Effects of prenatal DEX exposure (DEX), postnatal HF diet (VHF), and prenatal DEX exposure plus postnatal HF diet (DHF) on systolic blood pressure. Blood pressure was measured in conscious rats by an indirect tail-cuff method at 6 months of age. *N* = 4–6/group. A two-way ANOVA was used to evaluate the effect of prenatal DEX (Hit 1) and postnatal HF diet (Hit 2) and their interaction effect (Hit 1 x Hit 2). *compared with VEH, *p* < 0.05; ^#^compared with DEX, *p* < 0.05; ^§^compared with VHF, *p* < 0.05. Abbreviations: VEH, vehicle; DEX, prenatal dexamethasone exposure; VHF, postnatal high-fat diet exposure; DHF, prenatal dexamethasone exposure plus postnatal high-fat diet; SBP, systolic blood pressure

Two-way ANOVA revealed significant main effects for prenatal DEX exposure [F (1, 8)  =  71.25, *p* < 0.001] and postnatal HF diet [F (1, 8)  =  61.02, *p* <  0.001] on systolic BP (Fig. 3). The combined main effects of Hit 1 and Hit 2 resulted in the highest systolic BP in animals in the DHF, relative to that of vehicle (VEH) groups (189.40 ± 1.70 vs. 146.20 ± 2.92 mmHg). Thus, both prenatal GC exposure and postnatal HF have impacts on systolic BP. Prenatal DEX plus postnatal HF exerted a synergistic effect on the increase of systolic BP.

### Prenatal DEX exposure decreased plasma ANG I and ANG II levels, whereas postnatal HF diet decreased ANG-(1–7) level

**Fig. 4:**
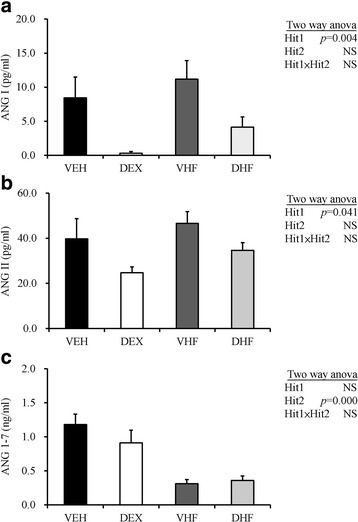
Effects of prenatal DEX exposure (DEX), postnatal HF diet (VHF), and prenatal dexamethasone exposure plus postnatal (DHF) on plasma angiotensin related peptides. The plasma levels of (**a**) angiotensin I, (**b**) angiotensin II, (**c**) angiotensin-(1–7) of rats with indicated treatment at 6 months of age. *N* = 8–9/group. Two-way ANOVA was used to evaluate the effect of prenatal DEX exposure (Hit 1), postnatal HF diet (Hit 2), and their interaction (Hit 1 x Hit 2). Abbreviations: VEH, vehicle; DEX, prenatal DEX exposure; VHF, postnatal HF diet; DHF, prenatal dexamethasone exposure plus postnatal HF diet. ANG I, angiotensin I; ANG II, angiotensin II; ANG-(1–7), angiotensin-(1–7)

We also determined the plasma levels of ANG I, ANG II, and ANG-(1–7) by using ELISA. As shown in Fig. 4a, only the effect of Hit 1 was significant for ANG I [F (1, 48) = 9.86, *p* = 0.004], but no effect of Hit 2 or interaction of the effects were observed. Prenatal DEX exposure resulted in the lowest plasma levels of ANG I in the DEX relative to VEH groups (0.29 ± 0.25 vs. 8.43 ± 3.05 pg/ml). For ANG II, similar to ANG I, only the effect of Hit 1 was significant [F (1, 30) = 4.57, *p* = 0.041], but no effect of Hit 2 or interaction of the effects was observed. Prenatal DEX exposure resulted in the lowest plasma levels of ANG II in the DEX relative to VEH group (24.69 ± 2.65 vs. 39.68 ± 8.99 pg/ml). However, postnatal HF diet resulted in significantly lower plasma ANG-(1–7) level than that in control animals fed with chow-diet [F (1, 31) = 29.67; *p* < 0.001] (Fig. 4c). There was no interaction between Hit 1 and Hit 2 in these animals. Although both prenatal DEX and postnatal HF resulted in elevation of systolic pressure, it seems they modulate RAS system through different mechanisms. Prenatal DEX exposure led to a decrease in plasma ANG I and ANG II levels, whereas postnatal HF diet caused the plasma ANG-(1–7) to decrease.

### The effects of prenatal DEX exposure and postnatal HF diet on the expression of RAS signaling genes in retroperitoneal fat

**Fig. 5:**
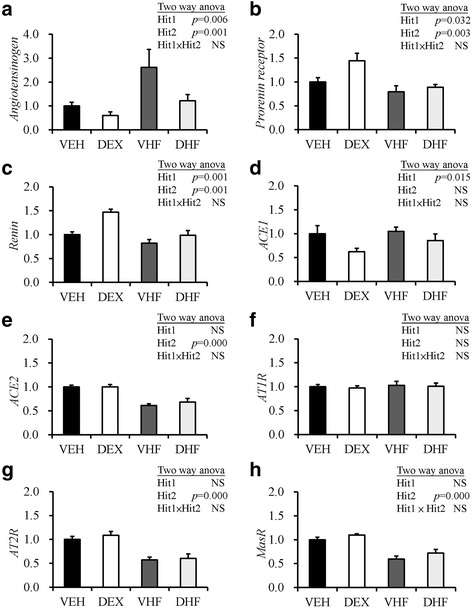
Effects of prenatal DEX exposure (DEX), postnatal HF diet (VHF), and prenatal dexamethasone exposure plus postnatal HF diet (DHF) on gene expressions of renin-angiotensin system in the retroperitoneal adipose tissue. **a** prorenin receptor, **b** renin, **c** angiotensinogen, **d** *ACE1*, **e** *ACE2*, **f** *AT1R*, **g** *AT2R*, and **h** *Mas R* mRNA expressions of retroperitoneal adipose tissue in 6-month-old rats with indicated treatment

We next analyzed the effect of prenatal DEX exposure and postnatal HF diet on the expression of RAS signaling genes. Many studies have shown that the expression of common reference genes varies with tissue type and physiological status [36]. In order to find a reliable reference gene for mRNA expression analysis, we investigated the expression of *cyclophilin A*, *glyceraldehyde 3-phosphate dehydrogenase (GAPDH)*, *ribosomal protein L17*, *β2 microglobulin*, and *β-actin* mRNAs. We found that *GAPDH* showed less variation than the other reference genes in rat adipose tissue (Additional file 2: Figure S1). Therefore, we used *GAPDH* as the reference gene in subsequent studies. As shown in Fig. 5a, prenatal DEX exposure suppressed the expression of *AGT* gene (0.60 ± 0.15-fold relative to the VEH group), whereas the VHF group showed increased expression of *AGT* mRNA in rat fat tissue (2.61 ± 0.75-fold relative to the VEH group). Two-way ANOVA revealed significant main effects for Hit 1 [F (1, 20)  =  9.51, *p* = 0.006] and Hit 2 [F (1, 20)  =  16.33, *p* =  0.001] but without significant interaction between Hit 1 and Hit 2. This result indicated that these two factors acted independently. In contrast to the effect on *AGT* mRNA, prenatal DEX exposure and postnatal HF enhanced and suppressed *prorenin receptor* mRNA expression, respectively (DEX vs. VEH, 1.44 ± 0.16-fold; VHF vs. VEH, 0.79 ± 0.13-fold) (Fig. 5b). Two-way ANOVA revealed significant main effects for Hit 1 [F (1, 20)  =  5.31, *p* = 0.032] and Hit 2 [F (1, 20)  =  11.68, *p* =  0.003] without significant interaction between Hit 1 and Hit 2. The effect on the expression of *renin* mRNA was like that of *prorenin receptor* mRNA. Prenatal DEX exposure and postnatal HF enhanced and suppressed *renin* mRNA expression, respectively (DEX vs. VEH, 1.47 ± 0.07-fold; VHF vs. VEH, 0.82 ± 0.08-fold) (Fig. 5c). Two-way ANOVA revealed significant main effects for Hit 1 [F (1, 20) = 14.20, *p* = 0.001] and Hit 2 [F (1, 20) = 15.62, *p* = 0.001] without significant interaction between Hit 1 and Hit 2. Regarding mRNA expression of *ACE1*, there was a significant effect of prenatal DEX treatment [F (1, 20) = 7.11; *p* = 0.015; Fig. 5d], but no effect of postnatal HF was observed, and there was no interaction. In contrast, mRNA expression of *ACE2* was significantly affected only by postnatal HF [F (1, 20) =41.92; *p* < 0.001; Fig. 5e], and no effect of Hit 1 or interaction of the effects were observed. There was no effect of either prenatal DEX exposure or postnatal HF on *AT1R* mRNA expression and there was no interaction of the effects (Fig. 5f). Two-way ANOVA between groups showed a significant main effect only for postnatal HF but not prenatal DEX exposure on the expression of *AT2R* mRNA [F (1, 20)  = 33.70, *p* < 0.001; Fig. 5g] without Hit 1/Hit 2 interactions. Similarly, in the case of *MasR* mRNA expression, only the effect of postnatal HF was significant [F (1, 20) =37.21; *p* < 0.001; Fig. 5h], but no effect of prenatal DEX or interaction of the effects were observed. Thus, prenatal DEX exposure and postnatal HF diet influenced the gene expressions of *prorenin receptor*, *AGT*, and *renin* of adipose tissue in an opposite direction, although both prenatal DEX exposure and postnatal HF diet cause elevation of systolic BP.

## Discussion

**Fig. 6:**
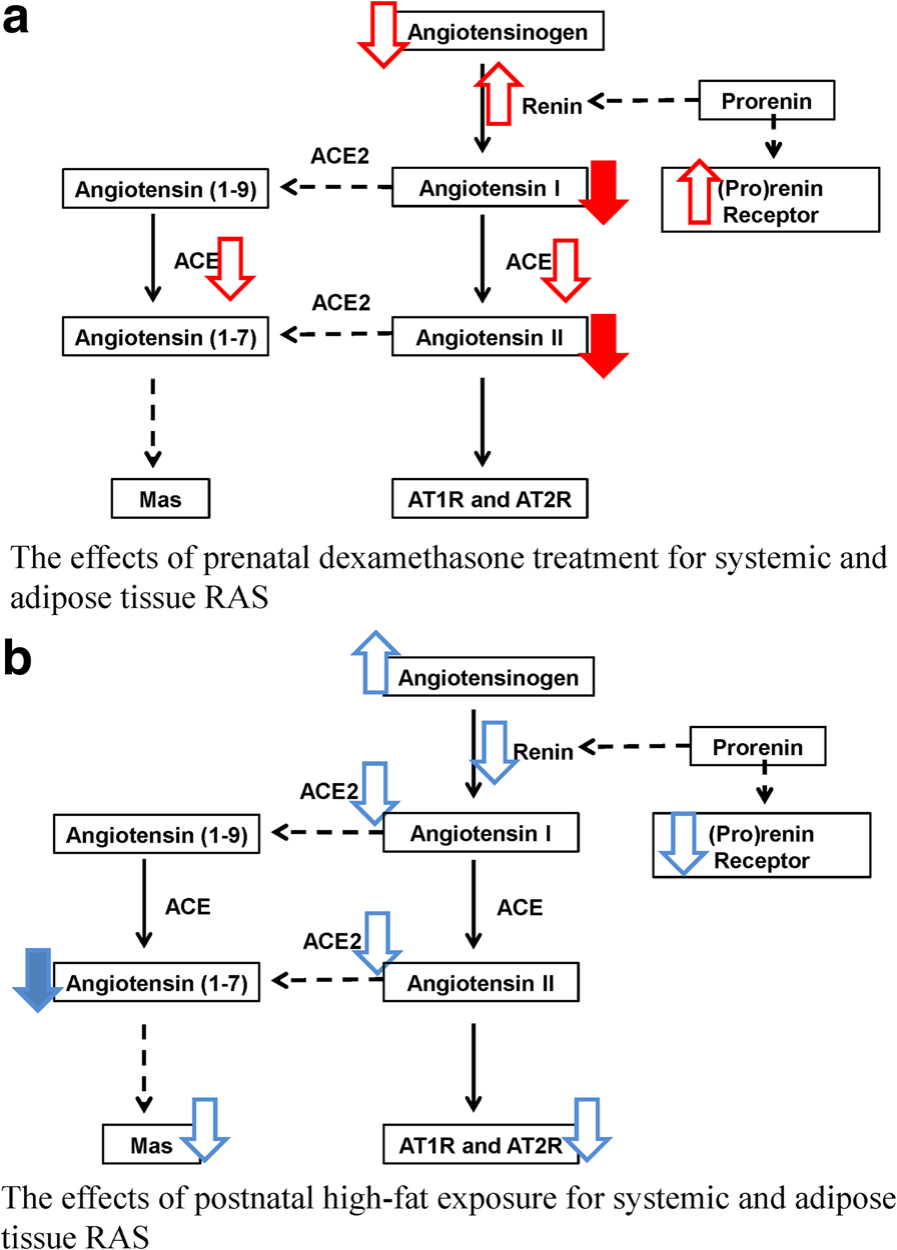
Effects of prenatal DEX exposure and postnatal HF diet on systemic RAS and systemic and adipose tissue RAS. (**a**) Prenatal DEX exposure tended to alter ACE1/ANG II/AT1R axis, whereas (**b**) postnatal HF diet tended to suppress ACE2/ANG-(1–7)/MasR axis suppression. Solid arrow head indicates systemic RAS change and hollow arrow head indicates adipose RAS change

In the present study, we used an animal model to study the impact of prenatal GC exposure and postnatal HF diet on BP, plasma ANG related peptides, and expression of genes involved in RAS signaling in adipose tissue. Both prenatal GC exposure and postnatal HF diet had an impact on systolic BP. In the case of plasma RAS peptide levels, prenatal GC exposure suppressed plasma ANG I and ANG II levels. Postnatal HF diet led to the suppression of plasma ANG-(1–7) level but did not impact ANG I and ANG II levels. Prenatal GC exposure enhanced the mRNA expression of *prorenin receptor* and *renin* and suppressed the mRNA expressions of *angiotensinogen* and *ACE* in adipose tissue. In contrast, postnatal HF diet upregulated the expression of the *angiotensinogen* mRNA and downregulated the expression of *prorenin*, *renin*, *ACE2*, *ATR2*, and *Mas* mRNAs in adipose tissue. We found prenatal GC exposure alter the ACE1/ANG II/AT1R axis, whereas postnatal HF diet negatively impacted the ACE2/ANG-(1–7)/MasR axis (Fig. 6). Thus, prenatal DEX exposure and postnatal HF diet had a synergistic effect on the elevation of BP through a distinct programming mechanism of systemic and adipose RAS.

RAS is known for its role in blood pressure regulation and fluid/electrolyte homeostasis. ANG-(1–7) treatment of rodents fed with HF diet improved the lipid metabolism, obesity, and hepatic inflammation, by downregulating resistin/TLR4/NF-κB pathway [37, 38]. In our study, we found that postnatal HF diet resulted in a decrease in plasma ANG-(1–7) levels. The decrease in plasma ANG-(1–7) levels might be an important mechanism for HF diet-induced obesity and inflammation. In addition to systemic RAS, local tissue-based ANG peptide formation and function, separately from the circulating RAS, have been detected in various tissues, including the kidneys, cardiovascular, reproductive, adipose and nervous system tissues [25, 39]. These local RAS have a variety of functions, including cardiovascular regulation, neurotransmission, electrolyte transport, and endocrine and fertility regulation, in association with or independently of the systemic RAS [39–41].

Adipose tissue has recently been viewed as an endocrine organ. Adipocyte homeostasis is essential to maintain the equilibrium between nutrient utilization and storage. Recent studies pointed out the potent contribution of adipose tissue’s RAS to systemic RAS and obesity related hypertension. Overexpression of adipose AGT in mice has been reported to induce hypertension and increase body fat and plasma AGT levels [42]. Adipocyte AGT deficiency has showed to prevent high fat-induced plasma ANG II and systolic BP increase in mice [43]. These results demonstrated the contribution of adipose derived AGT to BP regulation. However, in our study, lower *AGT* gene expression with prenatal DEX exposure did not rescue the systolic BP increase induced by postnatal HF. It suggests other skewed RAS signaling molecules and even other target organ are also important for the maintenance of blood pressure.

Studies in animals and humans have focused on the regulation of different RAS components of adipose tissue with respect to diet-induced obesity and hypertension. *AGT* mRNA and protein significantly increased in the adipose tissue of mice weaned onto a HF diet [44]. In humans, adipose *AGT* gene expression was shown to be elevated in obese subjects [45]. Postnatal HF diet can increase the number of pro-renin receptor expressing cells in adipose tissue in rats [46]. Our results from postnatal HF group agreed with these reports. In our study, postnatal HF diet increased *AGT* mRNA in the adipose tissue weaned onto a HF diet. Furthermore, decrease of *ACG2* mRNA and plasma Ang-(1–7) were also observed in our study. ACE2, which is the enzyme that cleaves ANG II into Ang-(1–7), protects against HF diet-induced insulin resistance in mice [47, 48]. In one study on mice fed with HF diet, adipose expression of *ACE2* mRNA increased [49]. But we found that the expression of *ACE2* mRNA in retroperitoneal adipose tissue decreased with long-term HF diet in rats. However, these results demonstrated that the expression of ACE2 in adipocytes was dysregulated in subjects fed with HF diet. Since Ang-(1–7) is cleaved from ANG II by ACE2, the decrease of plasma Ang-(1–7) in postnatal HF group may partly due to the less expression of *ACE2* mRNA in adipose tissue.

MasR is a G protein-coupled receptor and is associated with increased glucose uptake and insulin sensitivity [50]. Mice lacking MasR showed an increase in the abdominal fat mass, associated with higher adipose tissue AGT expression [51]. AT2R activation or deletion also influenced the HF diet-induced adiposity and obesity [52, 53]. The decrease in the expression of *MasR* and *AT2R* mRNAs in adipose tissue in the postnatal HF diet group in our study, suggested the dysregulation of adipose RAS cascade in HF diet related metabolic disorder.

GC was shown to upregulate the expression and secretion of AGT in adipose cells in a dose-dependent manner [54]. However, the evidence linking GC-related blood pressure increase and RAS is equivocal. Prenatal GC exposure in late gestation increased plasma renin activity in rat offspring during adulthood [55]. However, despite the higher MAP, there is no difference in renin, AGT, ACE, and ANG related peptides in sheep exposed to prenatal DEX had been reported. In our study, prenatal DEX treatment resulted in BP increase, but a decrease in plasma ANG I/II compared to that in VEH group. It suggested that the fetal programming effect in the development of hypertension by prenatal GC exposure, is partly due to systemic RAS independent mechanisms [56]. The role of local RAS in prenatal GC exposure related hypertension should also be considered. There are very few reports on the effect of prenatal GC exposure on the RAS in the adipose tissue of the offspring. One report showed that prenatal betamethasone treatment increased *ACE1* mRNA, but not AGT in adipose tissue of male offspring sheep [57]. In our study, we found that prenatal DEX treatment suppressed *ACE1* mRNA expression in retroperitoneal fat of rats. Thus, the effect of different GC regimens seems not the same in different species.

Our study has a few limitations. Renin and prorenin bind to their receptors and enhance the enzymatic activity of renin. Therefore, renin-dependent formation of angiotensin peptides is locally modulated by renin receptors in adipocytes. In this study, we determined the mRNA expression levels of *renin* and *prorenin receptor*, rather than the renin activity. Therefore, further studies focusing on the enzymatic activity of renin can provide insights into the modulatory functions of renin. Because many RAS molecules are released after their production, we have checked the plasma levels of ANG peptides to represent the abundance of RAS down-stream signaling. Determine the protein amount of indicated RAS molecules precisely in further study may help us to clarify the RAS is activated or inhibited. Another limitation of this study is only local RAS of adipose tissue is being investigated here. Other local RAS including vascular and kidney RAS may be influenced by prenatal DEX exposure and postnatal high-fat diet.

## Conclusions

Although both prenatal GC exposure and postnatal HF diet program contribute to the development of hypertension, their effects on systemic and local RAS are different. RAS inhibitors, including ACE inhibitors, ANG II receptor blockers, and renin inhibitors are commonly used in the treatment of hypertension. Medications aimed at systemic RAS also affect the local systems, beneficially or adversely [6]. Since postnatal HF diet resulted in higher systemic ANG peptides and AGT in adipose tissue, ACE inhibitors and ANG II receptor blockers may be more suitable treatments for HF diet associated hypertension. Prenatal GC exposure increased the gene level of *renin* but lowered the *ACE 1* mRNA and ANG peptide expressions. Therefore, renin inhibitors may be good candidates for prenatal GC exposure related hypertension. Our findings from this study provide new insights into the different mechanisms of hypertension programming, and provide specific RAS targets for the precise treatment of hypertension.

## Additional files

Additional file 1: **Table S1.** The primer sequences used for quantitative polymerase chain reaction (qPCR). (DOC 43 kb)

Additional file 2: **Figure S1.** The mRNA expressions of indicated reference genes in rat adipose tissue. Two micrograms cDNA was used for each sample and PCR was performed for *CYC*, *GAPDH*, *RPL17*, *B2M*, and β-actin. Among the five reference genes, *GAPDH* had the smallest coefficient of variance (CV). *N* = 3/group. Abbreviations: CYC, cyclophilin A; GAPDH, glyceraldehyde 3-phosphate dehydrogenase; RPL17, Ribosomal protein L17; B2M, β2 microglobulin. (TIFF 590 kb)

## Abbreviations

ACE: Angiotensin-converting-enzyme
ACE1: Angiotensin-converting-enzyme 1
ACE2: Angiotensin-converting-enzyme 2
AGT: Angiotensinogen
ANG: Angiotensin
ANG1: Angiotensin 1
ANG2: Angiotensin 2
ATR1: ANG II type 1 receptor
ATR2: ANG II type 2 receptor
DEX: Dexamethasone
GAPDH: Glyceraldehyde 3-phosphate dehydrogenase
GC: Glucocorticoid
HF: High-fat
MasR: Mas receptor
RAS: Renin–angiotensin system
