# Supplementary material for: Prenatal dexamethasone and postnatal high-fat diet have a synergistic effect of elevating blood pressure through a distinct programming mechanism of systemic and adipose renin–angiotensin systems
Source: Lipids Health Dis. 2018 Mar 14;17:50. doi: 10.1186/s12944-018-0701-0 (PMC5853160; doi:10.1186/s12944-018-0701-0)
Supplement: Supplementary file 1 — Table S1. The primer sequences used for quantitative polymerase chain reaction (qPCR). (DOC 43 kb) [file 12944_2018_701_MOESM1_ESM.doc]

Additional file 1: Table S1. The primer sequences used for quantitative polymerase chain reaction (qPCR)

Primer sequences for qPCR

| *Renin* | sense | 5'- AACATTACCAGGGCAACTTTCACT -3' |
| --- | --- | --- |
|  | antisense | 5'- ACCCCCTTCATGGTGATCTG -3' |
| *Prorenin receptor* | sense | 5'- GAGGCAGTGACCCTCAACAT -3' |
|  | antisense | 5'- CCCTCCTCACACAAGAAGGT -3' |
| *AGT* | sense | 5'- GCCCAGGTCGCGATGAT -3' |
|  | antisense | 5'- TGTACAAGATGCTGAGTGAGGCAA -3' |
| *ACE* | sense | 5’- CACCGGCAAGGTCTGCTT -3’ |
|  | antisense | 5’- CTTGGCATAGTTTCGTGAGGAA -3’ |
| *ACE2* | sense | 5’- GCCAGGAGATGACCGGAAA -3’ |
|  | antisense | 5’- CTGAAGTCTCCATGTCCCAGATC -3’ |
| *AT1R* | sense | 5’- GCTGGGCAACGAGTTTGTCT -3’ |
|  | antisense | 5’- CAGTCCTTCAGCTGGATCTTCA -3’ |
| *AT2R* | sense | 5’- CAATCTGGCTGTGGCTGACTT -3’ |
|  | antisense | 5’- TGCACATCACAGGTCCAAAGA -3’ |
| *Mas R* | sense | 5’- CATCTCTCCTCTCGGCTTTGTG -3’ |
|  | antisense | 5’- CCTCATCCGGAAGCAAAGG -3’ |
| *B2M* | sense | 5’- TGACCGTGATCTTTCTGGTG -3’ |
|  | antisense | 5’- ATCTGAGGTGGGTGGAACTG -3’ |
| *CYC* | sense | 5’- CCCACCGTGTTCTTCGACAT -3’ |
|  | antisense | 5’- CCAGTGCTCAGAGCACGAAA -3’ |
| *RPL17* | sense | 5’- CCAGGTGAACAAGGCTCCTA -3’ |
|  | antisense | 5’- AATTTATTCCCGTGCCATGA -3’ |
| *β-actin* | sense | 5'- TACTGCCCTGGCTCCTA -3' |
|  | antisense | 5'- GGGCCGGACTCATCGTA -3' |
| *GAPDH* | sense | 5'- TCTTGTGCAGTGCCAGCCTC -3' |
|  | antisense | 5'- GTCACAAGAGAAGGCAGCCCTGG -3' |

Abbreviation: B2M; β-2-icroglobulin, CYC; Cyclophilin, RPL17; Ribosomal protein L17, AGT; Angiotensinogen
