# Supplementary figures and images for: Prenatal dexamethasone and postnatal high-fat diet have a synergistic effect of elevating blood pressure through a distinct programming mechanism of systemic and adipose renin–angiotensin systems
Source: Lipids Health Dis. 2018 Mar 14;17:50. doi: 10.1186/s12944-018-0701-0 (PMC5853160; doi:10.1186/s12944-018-0701-0)

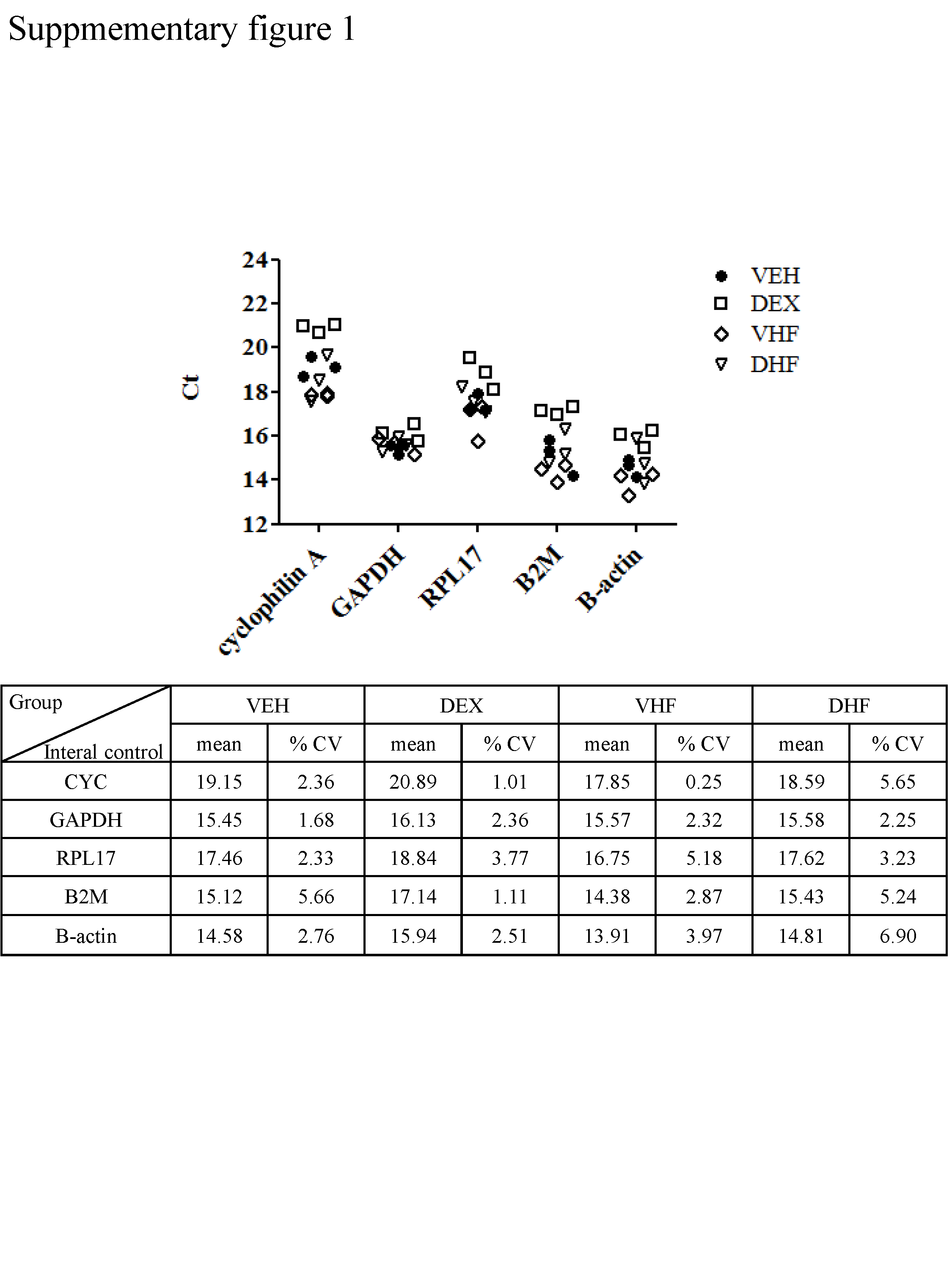

Supplement: Supplementary file 2 — Figure S1. The mRNA expressions of indicated reference genes in rat adipose tissue. Two micrograms cDNA was used for each sample and PCR was performed for CYC, GAPDH, RPL17, B2M, and β-actin. Among the five reference genes, GAPDH had the smallest coefficient of variance (CV). N = 3/group. Abbreviations: CYC, cyclophilin A; GAPDH, glyceraldehyde 3-phosphate dehydrogenase; RPL17, Ribosomal protein L17; B2M, β2 microglobulin. (TIFF 590 kb) [file 12944_2018_701_MOESM2_ESM.tiff]
